# Lung Extracellular Matrix Hydrogels Enhance Preservation of Type II Phenotype in Primary Alveolar Epithelial Cells

**DOI:** 10.3390/ijms23094888

**Published:** 2022-04-28

**Authors:** Esther Marhuenda, Alvaro Villarino, Maria Leonor Narciso, Marta Camprubí-Rimblas, Ramon Farré, Núria Gavara, Antonio Artigas, Isaac Almendros, Jorge Otero

**Affiliations:** 1 Unitat de Biofísica i Bioenginyeria, Facultat de Medicina i Ciències de la Salut, Universitat de Barcelona, 08036 Barcelona, Spain; marhuenda.esther@gmail.com (E.M.); alvaro.villarino.romero@gmail.com (A.V.); mnarciso@ibecbarcelona.eu (M.L.N.); rfarre@ub.edu (R.F.); ngavara@ub.edu (N.G.); ialmendros@ub.edu (I.A.); 2 CIBER de Enfermedades Respiratorias, 28029 Madrid, Spain; mcamprubi@tauli.cat (M.C.-R.); aartigas@tauli.cat (A.A.); 3 The Institute for Bioengineering of Catalonia (IBEC), The Barcelona Institute of Science and Technology (BIST), 08028 Barcelona, Spain; 4 Corporació Sanitària Universitària Parc Tauli, I3PT, Universitat Autònoma de Barcelona, 08193 Sabadell, Spain; 5 Institut d’Investigacions Biomèdiques August Pi i Sunyer, 08036 Barcelona, Spain

**Keywords:** extracellular matrix, hydrogels, alveolar cells, type II phenotype, YAP

## Abstract

One of the main limitations of in vitro studies on lung diseases is the difficulty of maintaining the type II phenotype of alveolar epithelial cells in culture. This fact has previously been related to the translocation of the mechanosensing Yes-associated protein (YAP) to the nuclei and Rho signaling pathway. In this work, we aimed to culture and subculture primary alveolar type II cells on extracellular matrix lung-derived hydrogels to assess their suitability for phenotype maintenance. Cells cultured on lung hydrogels formed monolayers and maintained type II phenotype for a longer time as compared with those conventionally cultured. Interestingly, cells successfully grew when they were subsequently cultured on a dish. Moreover, cells cultured on a plate showed the active form of the YAP protein and the formation of stress fibers and focal adhesions. The results of chemically inhibiting the Rho pathway strongly suggest that this is one of the mechanisms by which the hydrogel promotes type II phenotype maintenance. These results regarding protein expression strongly suggest that the chemical and biophysical properties of the hydrogel have a considerable impact on the transition from ATII to ATI phenotypes. In conclusion, culturing primary alveolar epithelial cells on lung ECM-derived hydrogels may facilitate the prolonged culturing of these cells, and thus help in the research on lung diseases.

## 1. Introduction

The fundamental questions on the precise mechanisms underlying alveolar epithelial cells (AECs) damage and epithelium repair in relevant diseases, such as acute respiratory distress syndrome or chronic obstructive pulmonary disease, are still unsolved. Although it is well-known that the alveolar epithelium is repaired by the proliferation of type II AECs (ATIIs), which differentiate into type I phenotype cells (ATIs), the involved mechanisms are still poorly understood [1]. In fact, a limitation hampering translational studies in lung diseases is the difficulty of maintaining the type II phenotype of primary AECs in vitro. Indeed, it is widely known that ATII-to-ATI transdifferentiation occurs very quickly in vitro; thus, primary type II AECs neither adequately proliferate, nor can be subcultured under conventional culture conditions [2].

To extend the maintenance of the type II AECs proliferative phenotype in vitro, it was proposed to coat the culture plates with hydrogels, such as Matrigel, resembling the extracellular matrix (ECM) [3]. Moreover, pioneering studies on subculturing strategies for primary ATII cells [4] employed inhibitors of Rho kinases, as the connection between the activation of the Rho pathway and cell mechanosensing of the ECM is well-established [5]. However, although the relationship between ATII phenotype maintenance in vitro and culturing cells on ECM-like substrates was proposed three decades ago [6], the problem remains open, probably because no hydrogels derived from lung ECM were available.

Interestingly, based on an initial report for obtaining hydrogels from the ECM of decellularized lungs [7], we have recently described a procedure for preparing such hydrogels by exclusively using lung ECM, with no need to add potentially toxic external cross-linkers [8]. If used as a culture substrate, this hydrogel, which realistically mimics the native lung ECM, could be particularly well-suited for providing a physiomimetic microenvironment to primary AECs. Therefore, we hypothesized that a lung ECM-based hydrogel would slow down the ATII-to-ATI transdifferentiation mediated by the inhibition of the Rho pathway, resulting in a decrease in F-actin polymerization and the formation of focal adhesions, as well as nuclear YAP activation [9,10,11].

## 2. Results

### 2.1. Primary Alveolar Epithelial Cells Form Monolayers on Lung-Derived Hydrogels

**Figure 1:**
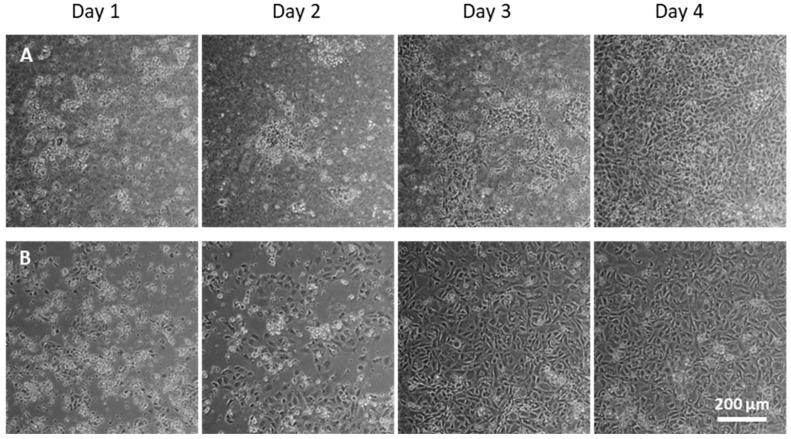
Rat primary alveolar cells were cultured either on porcine lung-derived hydrogel (**A**) or on a plate (**B**). Bright field images were taken every 24 h from day 1.

**Figure 2:**
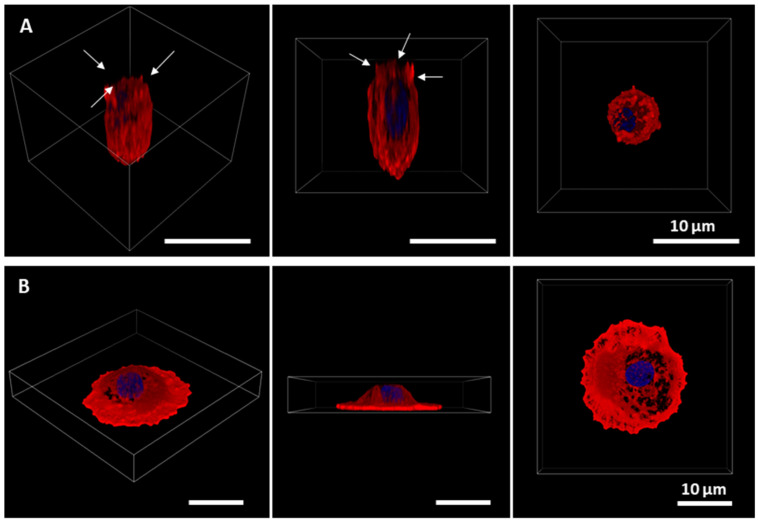
Three-dimensional representation of primary alveolar type II cells cultured for three days on hydrogel (**A**) and on a plate (**B**). Nuclei are stained in blue and actin cytoskeleton in red. In the case of the hydrogel-cultured cell, it can be distinguished by a cuboidal morphology and the presence of microvilli, indicated by arrows. In the case of the cell cultured on a plate, a more spread out and wider cytoplasm can be observed showing increased size.

Isolated AECs were grown either in lung-derived hydrogels or plates for four days. Cells were able to form a monolayer on a lung-derived hydrogel, as shown in bright-field images in Figure 1. Differences in the morphology of the cells as a function of the substrate were also noticeable as shown by confocal images in Figure 2. Throughout the culture, cells on plates started to show a more flattened shape as well as larger cytoplasms (cells cultured on plates presented, in general, sizes about 5 times larger than cells cultured on hydrogels), and the presence of vacuoles was noticeable. On the contrary, AECs cultured on hydrogels showed cuboidal shapes, and even their microvilli, a phenotypical characteristic of ATII cells [12], could be distinguished. Cells cultured on plates formed monolayers faster (day 3 vs. day 4).

### 2.2. Culture of Alveolar Epithelial Cells on Lung-Derived Hydrogels Preserves the Expression of Type II Markers for Longer Periods

**Figure 3:**
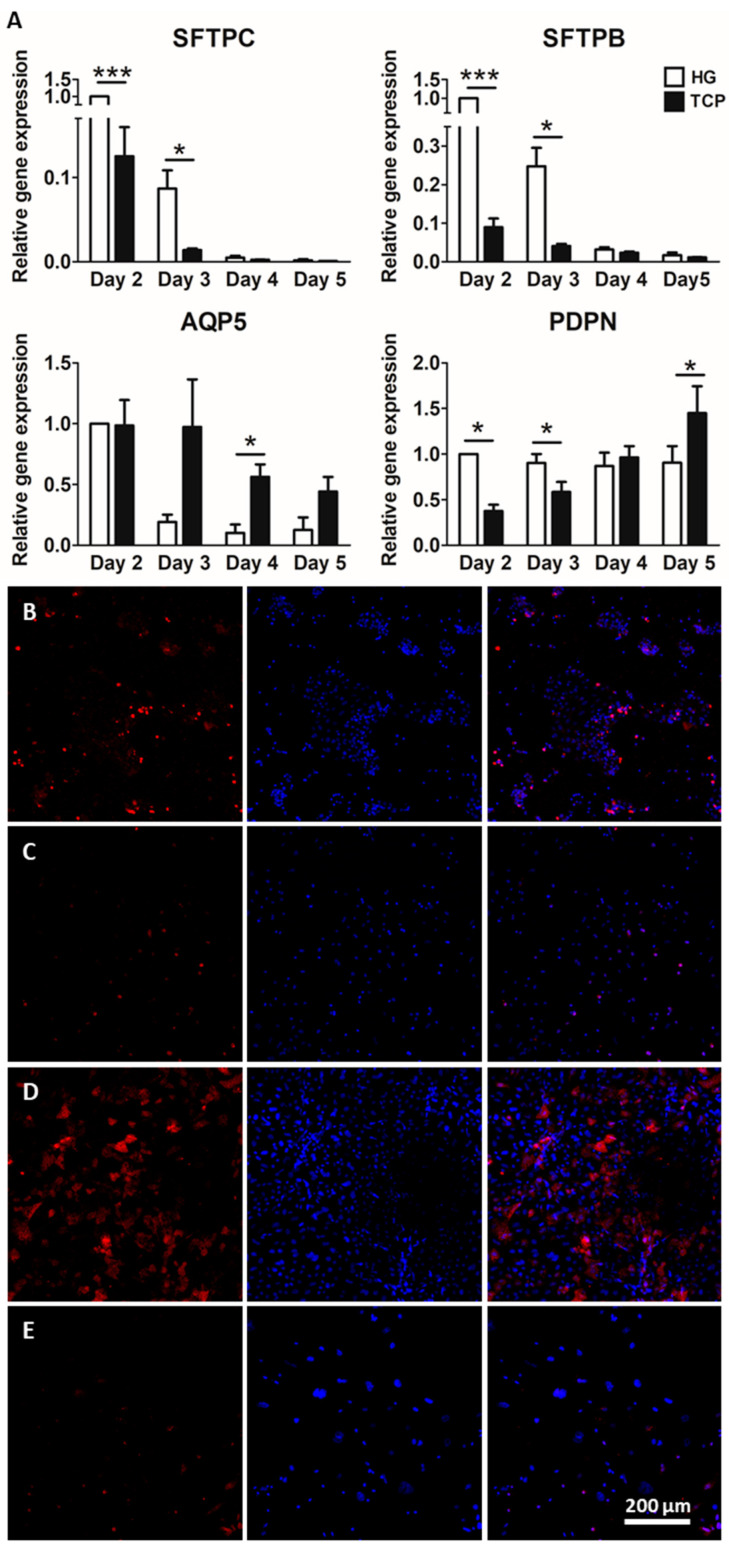
ATII cells were cultured either on hydrogel (HG) or a tissue culture plate (TCP). ATII typical marker surfactant proteins C (*sftpc*) and B (*sftpb*) and ATI typical marker aquaporin 5 (*aqp5*), podoplanin (*pdpn*) were studied at different time points (from day 2 to day 5) by qPCR (**A**). Relative gene expression is shown. Expression of surfactant protein C (SPC) was studied using immunofluorescence on ATII cells cultured on hydrogel at day 2 (**B**) and day 4 (**D**) and on a plate at day 2 (**C**) and day 4 (**E**). * *p* < 0.05, *** *p* < 0.001.

The results of the genetic expression of ATI and ATII typical markers (from day 2 to 5) are shown in Figure 3A. The gene expression of *sftpc* and *sftpb* decreased over time. There was a significant increase in ATII markers (surfactant proteins B and C) at earlier times (day 2 and day 3) in cells cultured on lung-derived hydrogels. There was no increase in ATI markers over time in cells cultured on hydrogels, in contrast to cells cultured on plates, where the expression of *pdpn* and *aqp5* increased with time (classical ATI markers). These results show that the ATII phenotype and gene expression levels were maintained for longer in lung-derived hydrogels. The results from immunostaining are shown in Figure 3B–E, revealing that the differential expression of surfactant protein C caused by the substrate was noticeable not only at the gene expression level, but also at the protein level. SPC expression was higher in cells cultured on lung hydrogels compared to that of cells cultured on a plate, as shown by the immunostainings. Furthermore, culture time affected cells differently depending on the substrate they were cultured on. Indeed, cells cultured on lung hydrogel were able to maintain the SPC expression over time, whereas cells cultured on a plate rapidly lost this ability.

### 2.3. Culture of Primary Alveolar Epithelial Cells in Lung-Derived Hydrogels Inhibits Type II-to-Type I Transdifferentiation by Altering the Hippo/Rho Pathway

**Figure 4:**
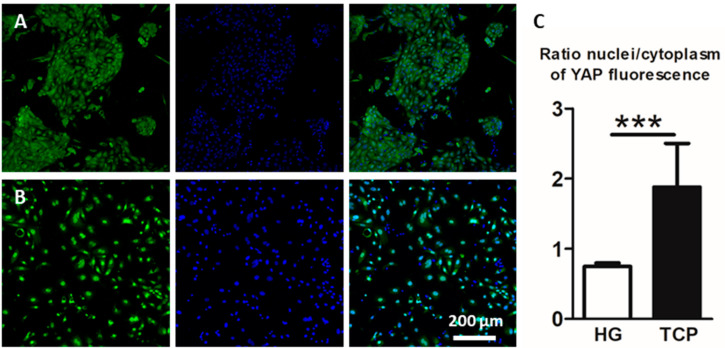
Alveolar type II cells were cultured on hydrogel (**A**) or on a plate (**B**) and stained for YAP protein (green). YAP nuclear and cytoplasmic expressions were quantified and expressed as the nuclei/cytoplasm fluorescence ratio in both conditions hydrogel (HG) and tissue culture plate (TCP) (**C**), *** *p* < 0.001.

Since YAP is a key mechanotransduction protein, its expression was studied in cells cultured on hydrogels or plates for three days (Figure 4A,B). In cells cultured on hydrogels, the YAP protein was located mainly in the cytoplasm, which indicates that it was being phosphorylated and subsequently degraded. In contrast, in cells cultured on plates, nuclear active YAP was observed. Specifically, there was a two-fold increase in the amount of nuclear YAP when cells were cultured on a plate compared to that of cells cultured on hydrogels, indicating a higher transcriptional activity in the first group (Figure 4C).

**Figure 5:**
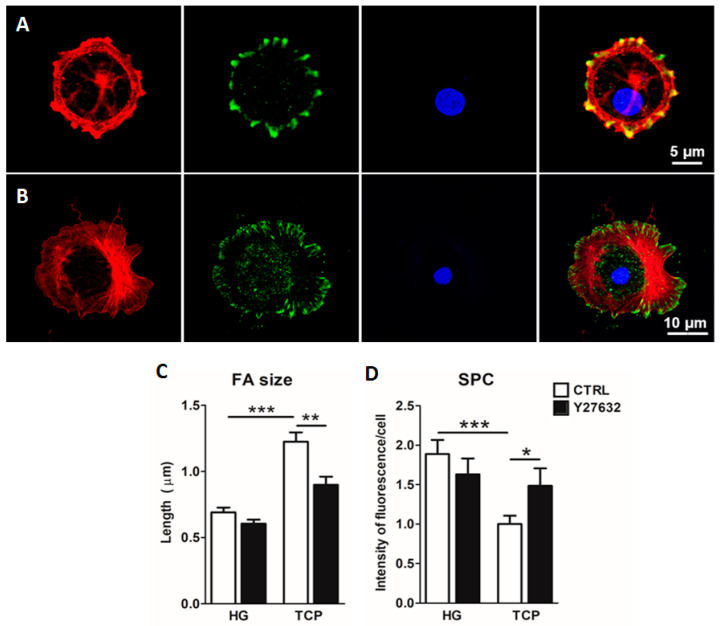
Representative images of focal adhesions (FAs) in cells cultured on hydrogels (**A**) and plate (**B**). Red: phalloidin, green: paxillin, blue: nuclei. Quantification of the FA length (**C**) and the intensity of surfactant protein C (SPC) of alveolar type II cultured on hydrogels for 3 days with (Y27632) and without (CTRL) the addition of the ROCK inhibitor (**D**), * *p* < 0.05, ** *p* < 0.01, *** *p* < 0.001.

Owing to the role of focal adhesions (FAs) and the actin cytoskeleton in sensing extracellular matrix cues and transmitting them to the cell, the expressions of actin and paxillin, which is one of the proteins comprising FAs, were studied. Both were reported to be implicated in the hippo pathway, by inhibiting it and promoting the YAP nuclear expression. In cells cultured for three days on lung-derived hydrogels, a poor focal adhesion assembly was observed by the paxillin immunostaining. Moreover, no stress fibers were formed as indicated by the phalloidin staining (Figure 5A). On the contrary, in cells that were cultured for three days on a plate, assembled paxillin and stress fibers were clearly observed (Figure 5B). The role of Rho, which is involved in the maturation of focal adhesions and YAP regulation [13,14] was studied by the use of the ROCK inhibitor (Y27632). The results show that it could play a role in the maintenance of the ATII phenotype, as reflected by an increase in SPC in conventional culture (*p* = 0.02) (Figure 5D) together with a decrease in focal adhesion size (*p* = 0.001) (Figure 5D), suggesting that the use of HGs as a substrate for AECs culture could be inhibiting the Rho pathway, and thus allowing for type II phenotype maintenance.

### 2.4. Primary Alveolar Epithelial Cells Cultured on Lung-Derived Hydrogels Can Be Subcultured

**Figure 6:**
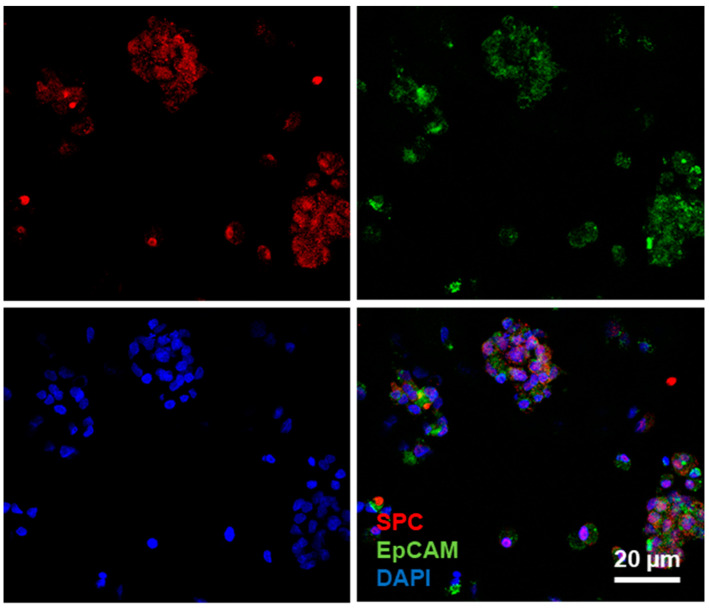
Alveolar type II cells were cultured on hydrogels for three days. After that time, they were subcultured on plates and stained for surfactant protein C (red) and EpCAM (green).

The potential ability to subculture primary ATII cells was studied. Cells were cultured for 3 days on lung hydrogels and, after that time, lung-derived hydrogels were digested with collagenase, and cells were seeded again on plates. Sixteen hours later, they were immunostained for typical epithelial (EpCAM) and ATII (SFTPC) markers (Figure 6). Subcultured cells were positive for both, EpCAM and SFTPC markers. These results indicate that lung-derived hydrogels allow for primary AECs subculture.

## 3. Discussion

We have provided evidence that using lung-derived ECM hydrogels as a culture substrate allows the maintenance of the type II phenotype in primary AECs to be enhanced. Moreover, our results for YAP and when using ROCK inhibitors on cells cultured on lung hydrogels, which preserve several proteins from their native organs [7], suggest that the biophysical properties of the hydrogel have a high impact on cell mechanosensing pathways, thereby playing a role in the maintenance of type II phenotype. This first-time study culturing cells on lung ECM-derived hydrogels, which was carried out in rat primary AECs as a proof of concept, opens the door for further research in primary human AECs, with a potential future impact on cell therapies for diseases such as pulmonary fibrosis and acute lung injury [15,16].

Similar results to those obtained in the present study were provided by Shannon and coworkers [6], who conducted the first study showing the importance of the protein content of the substrate in the maintenance of ATII morphology and phenotype. The main limitation of that first study was the inability of the cells to form a monolayer, which is easily accomplished in our lung-derived hydrogel. Since then, efforts have been made to improve the ability to maintain the ATII phenotype, which has only been achieved by media supplementation [3,17,18,19]. Both the biochemical and physical properties of the substrate are involved in cell behavior. It is worth noting that cells formed a monolayer slower in hydrogels than on plates, which could be due to the fact that cells proliferate quicker on the plate or that the ATII-to-ATI transdifferentiation allows for a faster substrate coverage, as ATI cells are much larger in size. In view of the differing stiffness of the hydrogels when compared to the culture plate and the cuboidal morphology of the cells, differences in their cytoskeleton distribution and the formation of focal adhesions, which are key points in the crosstalk of cell-ECM, were expected. Cells cultured on hydrogels showed shorter focal adhesions that correlated with a decreased YAP translocation to the nuclei. YAP is an important transcription factor that is implied in the regulation of several genes, and it is reported to be associated with inflammatory and epithelial damage situations in vivo where the ATII-to-ATI transdifferentiation is required [20,21]. In this case, YAP nuclear location of cells cultured on a plate could be triggering the differentiation in the ATI cell population. As Rho is implicated in the maturation of focal adhesions [14], and previous authors have pointed to it as a key factor in the transdifferentiation process [4,22], its role was studied. The determination of the individual contribution of different Rho proteins would be highly interesting. However, as it would be complex [14], we studied this effect through its inhibition using Y27632. The obtained results support the implication of Rho in the ATII phenotype, as its inhibition produced an increase in SPC and decrease in FAs only in cells cultured on a plate. The origin of this inhibition of the Rho pathway, although out of the scope of the present work, is probably related to the complex molecular composition of lung-derived hydrogels and their biomechanical properties.

Primary ATII cells have previously been subcultured [4] by coculturing them with fibroblasts as feeder cells and with the addition of the Rho inhibitors. The role of fibroblasts in maintaining the ATII phenotype is not exactly known but it could be that they work as a source of the keratinocyte growth factor (KGF). The addition of KGF has been used by other authors to increase the phenotypic features of ATII cells [3,19], and in turn, to inhibit Rho kinases, which are shown to play a key role in the ATII-to-ATI transdifferentiation [22]. In our case, the use of lung-derived hydrogels as substrates for culturing allows these cells to be subcultured without the of use of additional chemicals or cocultures. Therefore, culturing primary alveolar epithelial cells on lung ECM-derived hydrogels may facilitate the prolonged culturing of these cells, and thus help in the research of lung diseases. However, more research should be carried out regarding the number of possible passages, especially considering that factors different from the ATI-to-ATII transdifferentiation would limit the time that these primary cells could be maintained in vitro. Further research is also needed to determine which components of ECM-derived hydrogels have more impact on the maintenance of the type II phenotype in vitro. As the extracellular matrix used to produce hydrogels in the present work was obtained from lung tissue, it is expected that some key biochemical and biophysical factors specific to the lung are being preserved in the process of hydrogel production. The identification of these factors, although out of the scope of the present work, would allow the development of novel optimized scaffolds for the in vitro culture of pulmonary cells.

## 4. Materials and Methods

Unless otherwise specified, all reagents were purchased from ThermoFisher Scientific (Waltham, MA, USA) or Sigma-Aldrich (Saint Louis, MO, USA).

### 4.1. Decellularized Lung Extracellular Matrix Hydrogels Preparation

Lung-derived hydrogels were prepared by following a previously described protocol [23]. Briefly, porcine lungs were decellularized by consecutive perfusion of the following reagents through the vasculature and the airways: 0.1% Triton X-100 and 2% SDC for 24 h at 4 °C, and NaCl 1 M and DNase solution for 1 h at 4 °C. Three washes of miliQ water were performed between consecutive reagent perfusion, and a last wash of PBS 1X was carried out. Decellularized lungs were cut into small pieces, freeze-dried (Telstar Lyoquest-55 Plus, Terrassa, Spain) and milled in liquid N_2_ (SPEX SamplePrep, Metuchen, NJ, USA). The obtained powder was resuspended at 20 mg/mL in 0.01 M HCl and pepsin digested at a 1/10 proportion under magnetic stirring at room temperature for 16 h. To produce hydrogels, the digested solution was pH-adjusted to 7.4 ± 0.4 by using 0.1 M NaOH and incubated at 37 °C for 20 min.

### 4.2. Primary Alveolar Epithelial Cells Isolation

The procedure was approved by the Ethical Board for Animal Research of the University of Barcelona, in compliance with regional, national and European regulations. Rat lungs were obtained from 180–250 g Sprague Dawley male rats. The animals were intraperitoneally anesthetized with 1 g/kg urethane and euthanized by exsanguination. AECs were isolated following a protocol described in [24]. Briefly, lungs were perfused with saline through the pulmonary artery and were resected en bloc. Three bronchioalveolar lavages were performed with 10 mL of PBS 1X to remove alveolar macrophages. Then, lungs were digested with 50 mL of 0.25% of trypsin through the airways for 30 min, cut into small pieces, and digested in a 100 units/mL DNase solution. The resulting suspension was filtered through a 100 µm and a 40 µm mesh and centrifuged through a Percoll (GE HealthCare, Chicago, IL, USA) gradient at 500× *g* for 20 min. The band containing ATII cells was recovered and digested using 20 units/mL of DNase. The resulting solution was centrifuged for 15 min at 500× *g*, and the pellet was resuspended in DCCM-1 (Biological Industries, Kibbutz Beit Haemek, Israel) medium and cultured for 1 h. Subsequently, medium containing ATII non-adherent cells was recovered and centrifuged for 10 min at 800× *g*. Cells were finally counted and seeded either on 24-well culture plates or on top of lung-ECM hydrogels.

### 4.3. Rho Kinase Inhibition Assay

For the inhibition of the Rho pathway, Y27638 (10 µM) was added to the medium for 24 h.

### 4.4. Reverse Transcription qPCR

For studying the expression of ATI and ATII markers, cells were cultured either on plastic or lung-derived hydrogels for different times, and RNA was subsequently extracted from samples by employing the RNeasy kit (Qiagen, Hilden, Germany). The cDNA was obtained by a reverse transcription-polymerase chain reaction (TaqMan Reverse Transcription Reagents, Invitrogen, Waltman, MA, USA) according to the manufacturer’s instructions. The expression level of surfactant protein C (*sftpc*), surfactant protein B (*sftpb*), aquaporin 5 (*aqp5*) and podoplanin (*pdpn*) was studied using the Taqman Fast Advanced Master Mix and the TaqMan Gene Expression Assays in a StepOnePlus thermocycler (Applied Biosystems, Waltham, MA, USA). The expression level of genes was normalized to the constitutively expressed gene PPIA and calculated using the 2^−ΔΔCt^ method [25].

### 4.5. Immunohistochemistry and Image Processing

For immunohistochemistry experiments, cells were fixed with 4% paraformaldehyde for 30 min. Primary antibodies were incubated overnight, and secondary antibodies were incubated for 2 h at 37 °C. Nuclei were stained with Hoechst 33342. To avoid unspecific binding, especially in the hydrogel samples, a blocking buffer consisting of 2% BSA (Thermo Fisher, MA, USA) diluted in PBS 1X (Gibco, MA, USA) was employed for 40 min. Primary antibodies employed were rabbit anti-SFTPC (Invitrogen, Waltman, MA, USA), mouse anti-YAP (Santa Cruz Biotechnology, Dallas, TX, USA) and EpCAM (Miltenyi, Bergisch Gladbach, Germany). Secondary antibodies used were goat anti-rabbit cy5 (Abcam, Cambridge, UK) and goat anti-mouse Alexa Fluor 488 (Abcam, Cambridge, UK). Images were acquired with a Nikon Confocal Eclipse Ti microscope using a 20 × Plan Fluor Multi-immersion objective (0.75 NA) in the case of the SFTPC, EpCAM and YAP staining with a 10× objective (0.3 NA) for the bright field images, and a 100× objective (1.45 NA) for the paxillin and actin images. Nuclear images were obtained at 450 nm when illuminating the sample at 408 nm. Samples were excited at 488 nm and acquired at 515 nm for YAP, EpCAM and Paxillin images, and excited at 543.5 nm and acquired at 605 nm for SFTPC and actin stains.

For the analysis of YAP images, five images per condition were randomly selected and analyzed using a blind procedure with ImageJ Software. Quantification of the ratio nuclear fluorescence/cytoplasmic fluorescence was assessed following a previously described procedure [26] with slight modifications. To calculate the total cell fluorescence, a triangle threshold was employed, and the integrated fluorescence was calculated in the YAP channel. For calculating the YAP nuclear fluorescence, the perimeter of the nuclei was delimited by the Huang threshold in the DAPI channel. After that, the resulting mask was redirected to the YAP channel and the integrated intensity contained in the nuclear perimeter was obtained. To calculate the cytoplasmic fluorescence, the differences in the intensities in the total cell and in the nuclei were calculated.

For focal adhesion length measurements, five representative adhesions per cell in paxillin stainings at the cell edge were manually quantified with Image J Software.

### 4.6. Statistical Analysis

Data are expressed as mean ± SE unless stated otherwise. Statistical analysis was performed with Graphpad Prism software. Differences in ATI/ATII markers gene expression and YAP nuclear/cytoplasmic expression were analyzed using paired *t*-test. A *p*-value < 0.05 was considered significant.

## Data Availability

Data supporting the findings of this study are available from the corresponding authors upon reasonable request.
